# Identification of miRNA Signatures during the Differentiation of hESCs into Retinal Pigment Epithelial Cells

**DOI:** 10.1371/journal.pone.0037224

**Published:** 2012-07-27

**Authors:** Ganlu Hu, Kevin Huang, Juehua Yu, Sailesh Gopalakrishna-Pillai, Jun Kong, He Xu, Zhenshan Liu, Kunshan Zhang, Jun Xu, Yuping Luo, Siguang Li, Yi E. Sun, Linda E. Iverson, Zhigang Xue, Guoping Fan

**Affiliations:** 1 Translational Center for Stem Cell Research, Tongji Hospital, Tongji University School of Medicine, Shanghai, China; 2 Stem Cell Research Center, Department of Regenerative Medicine, Tongji University School of Medicine, Shanghai, China; 3 Department of Human Genetics, David Geffen School of Medicine, University of California Los Angeles, Los Angeles, California, United States of America; 4 Stem Cell Biology, Beckman Research Institute of City of Hope, Duarte, California, United States of America; University of Southern California, United States of America

## Abstract

Retinal pigment epithelium (RPE) cells can be obtained through *in vitro* differentiation of both embryonic stem cells (ESCs) and induced pluripotent stem cells (iPSCs). We have previously identified 87 signature genes relevant to RPE cell differentiation and function through transcriptome analysis of both human ESC- and iPSC-derived RPE as well as normal fetal RPE. Here, we profile miRNA expression through small RNA-seq in human ESCs and their RPE derivatives. Much like conclusions drawn from our previous transcriptome analysis, we find that the overall miRNA landscape in RPE is distinct from ESCs and other differentiated somatic tissues. We also profile miRNA expression during intermediate stages of RPE differentiation and identified unique subsets of miRNAs that are gradually up- or down-regulated, suggesting that dynamic regulation of these miRNAs is associated with the RPE differentiation process. Indeed, the down-regulation of a subset of miRNAs during RPE differentiation is associated with up-regulation of RPE-specific genes, such as RPE65, which is exclusively expressed in RPE. We conclude that miRNA signatures can be used to classify different degrees of *in vitro* differentiation of RPE from human pluripotent stem cells. We suggest that RPE-specific miRNAs likely contribute to the functional maturation of RPE *in vitro*, similar to the regulation of RPE-specific mRNA expression.

## Introduction

The retinal pigment epithelium (RPE) is a polarized monolayer of pigmented cells between the neural retina and choroid. RPE plays many crucial roles in the eye including blood/retina barrier formation, nutrient transport between blood to photoreceptors, water conveyance from subretinal space to the blood, light absorption, growth factor secretion, and phagocytosis of the outer segment of photoreceptors [1]. For these reasons, dysfunction or death of RPE can lead to photoreceptor degeneration and eventual blindness in diseases such as Stargardt disease and aged related mascular degeneration (AMD) [2], [3], [4]. Although there are some treatments that partially alleviate symptoms of the wet form of AMD, there is no cure for AMD, particularly the more prevalent dry form of AMD [5].

Recent advances in stem cell biology have motivated new strategies to develop pluripotent stem-cell derived RPE for cell replacement therapy of AMD [6]. We and other labs have achieved the ability to generate functional RPE through *in vitro* differentiation of both human embryonic stem cells (hESCs) and induced pluripotent stem cells (hiPSCs) [7], [8], [9], [10], [11], [12]. However, the process can take between three to six months to achieve functional stem cell-derived RPE. Other approaches for promoting RPE differentiation treatment with defined factors or small molecules such as niconimide [7], [11], [13] However, our understanding of the molecular changes associated with RPE differentiation are still limited.

A recent microarray study comparing gene expression profiles of human fetal and adult RPE with somatic tissues identified 154 signature genes that are unique to RPE [14]. Following this study, we profiled mRNA expression during *in vitro* differentiation of hESCs/iPSCs into RPE [9]. By comparing the expression patterns of 154 RPE signature genes between hESCs/iPSCs versus stem cell-derived RPE, we refined the list of signature genes to a set of 87 RPE specific genes that can be used to monitor RPE differentiation and distinguish stem cell-derived RPE from other cell types.

In this current study, we followed the miRNA expression patterns during the course of RPE differentiation at four separate time points to survey molecular changes associated with RPE differentiation. MiRNAs are a class of 18∼22nt length small RNAs which can attenuate gene expression by inhibiting translation in the cytoplasm or promoting mRNA degradation in the nucleus [15]. There is growing evidence suggesting miRNAs play a role in RPE differentiation, survival, and function. For example, Dicer conditional knockout mice have decreased mature miRNA expression in retinas, leading to retinal degeneration and severely impaired visual function [16]. Therefore, elucidatingthe functional role of miRNAs during RPE differentiation will give us important insight into the crucial miRNAs involved in promoting RPE differentiation and maturation.

## Results

### RNA-seq analysis of miRNA profiles in human stem cells, RPE, and other types of somatic cells

We have developed a protocol to derive functional RPE cells from human pluripotent stem cells through a time window of three to six months or more [9]. Previously, we have demonstrated that stem cell-derived RPE are functionally equivalent to fetal RPEs and share global mRNA expression profile with cultured primary human RPE [9]. To profile miRNA expression patterns, we constructed small RNA libraries from 1) human ESCs, 2) 15-day partially differentiated hESCs, 3) early pigmented clusters (PC) that appear around 30 days, and 4) RPE cells in culture for over 3 months [9], [18]. Using high-throughput, we obtained a high-resolution miRNA profile in these cells. We also incorporated miRNA sequencing datasets of various human somatic tissues including heart, breast, skin and melanocytes procured from the Gene Expression Omnibus (GEO) database (Table S1).

**Figure 1:**
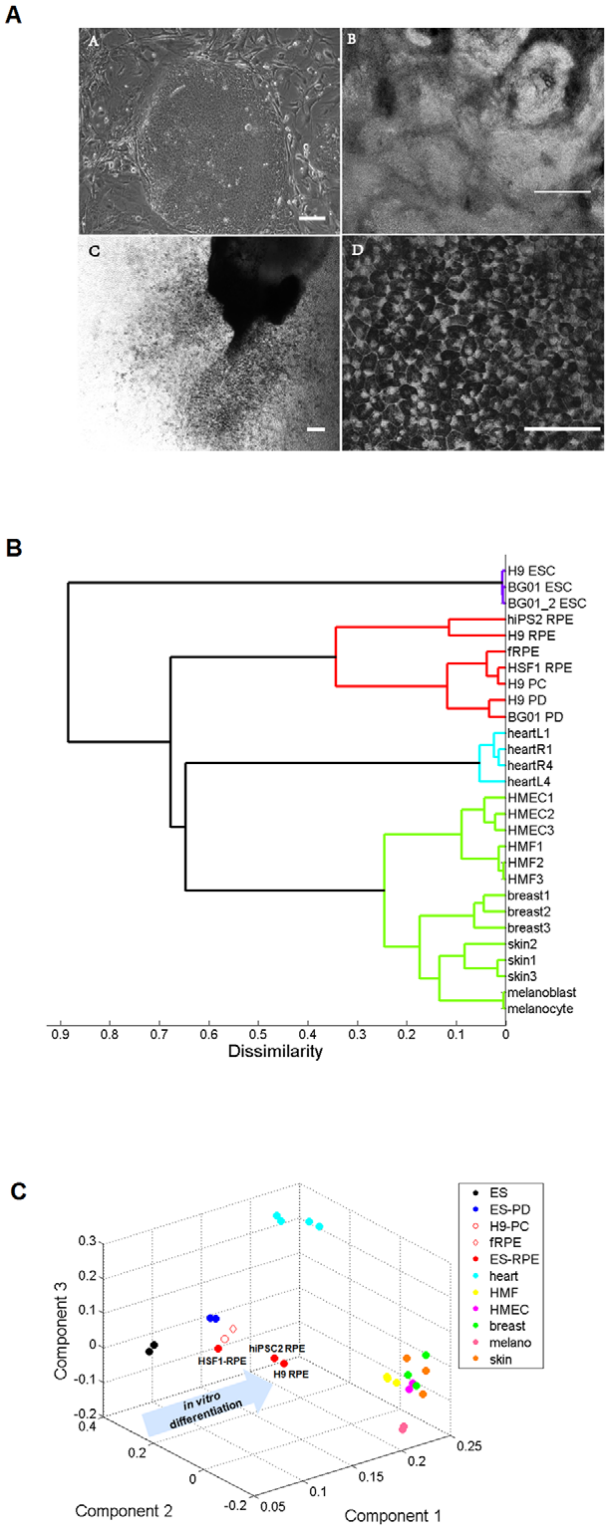
ES-derived RPE have distinctive global miRNA signatures. A) Morphological features of four stages during human pluripotent stem cells differentiate into RPE cells. a, human ES cells; b, partially differentiated ES cells after 4 days withdraw bFGF; c, pigment cluster appeared after about 30 days differentiation; d, RPE monolayer. Scare bar: (a–c) 100 μm; (b) 50 μm. B, C) MiRNA profiles of different cell types were clustered using either unbiased hierarchical clustering or principal component analysis (PCA). B) Pearson correlation clustering between all cell lines. C) 3D-biplot of the first three principal components. Various somatic tissues were downloaded from GEO database.

Unbiased hierarchical sample classification analysis and principal component analysis (PCA) both revealed distinct miRNA expression patterns in ES cells, partially differentiated cells, fully differentiated RPE cells, and other somatic tissues (Fig 1B, 1C). Interestingly, hierarchical clustering showed that HSF1-RPE and fetal RPE cluster closer to immature pigment clusters, indicating this particular batch of HSF1-RPE may not be fully mature compared to H9-RPE and hiPS2-RPE. In addition, our data indicated that partially differentiated hESCs and immature RPE shared many similar miRNA characteristics, consistent with the notion that miRNA signatures can be used to gauge the state of RPE differentiation. 3D-biplot of the first three principal components further confirmed our hypothesize that HSF1-RPE is immature compared to H9-RPE and hiPS2-RPE, which cluster away from ES and other partially differentiated cells and closer to fully differentiated somatic tissues. Importantly, RPE clustered away from melanoblast and melanocyte cells, another type of pigment cell also derived from neural crest. This is consistent with our previous study demonstrating distinct transcriptome differences between RPE and melanocytes [9], and suggests pigmentation between these two cell types may not be regulated in the same fashion or that the overall cell-type specific miRNA profile dominates the majority of differences between the two pigmented cell types. Moreover, we found RPE clustered away from other epithelial cell types, including breast and skin tissues and the human mammary epithelial cell line (HMEC), indicating RPE express a unique miRNA profile different from other epithelial cells. Collectively, our results revealed a distinct set of miRNAs for RPE compared to ES cells and other somatic tissues.

**Figure 2:**
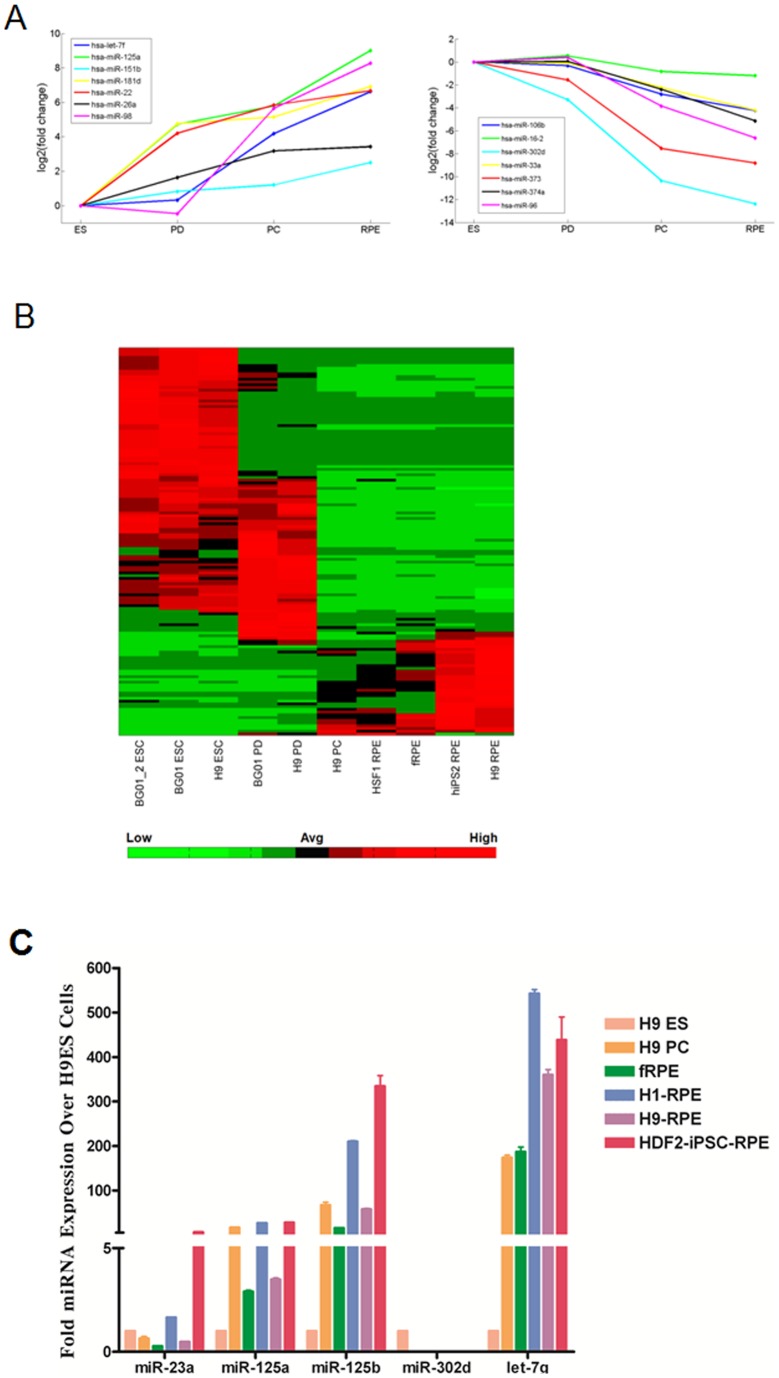
Select miRNAs are dynamically expressed during differentiation of H9 ES cell line. Representative miRNA expressions that are A) down-regulated or B) up-regulated during the course of differentiating H9 ESCs. All values were normalized to ES state, then log2 transformed. C) RT-PCR analysis of different RPE differentiation stage showing miR-23a, miR125a, miR-125b, miR-302d and let-7g expression relative to H9 ESC stage. The relative expression levels for each miRNA were normalized by hsa-U6 control. PD  =  partially differentiated, PC  =  pigmented clusters, ES  =  embryonic stem cells.

**Table 1:** Downregulated miRNAs in RPEs during ESC differentiation.

miRNA	H9 ESC	H9 RPE	log2(fold change)
hsa-miR-106b	2779	147	−4.2
hsa-miR-130a	1032	61	−4.1
hsa-miR-130b	521	19	−4.8
hsa-miR-1323	106	1	−6.4
hsa-miR-15a	166	30	−2.5
hsa-miR-15b	277	15	−4.2
hsa-miR-16-2	549	240	−1.2
hsa-miR-17	5685	694	−3.0
hsa-miR-18a	1387	44	−5.0
hsa-miR-25	440	84	−2.4
hsa-miR-301a	88	6	−3.8
hsa-miR-302c	9948	1	−13.3
hsa-miR-302d	6481	1	−12.4
hsa-miR-32	236	11	−4.5
hsa-miR-33a	62	3	−4.2
hsa-miR-363	3845	21	−7.5
hsa-miR-373	450	1	−8.8
hsa-miR-374a	2329	66	−5.1
hsa-miR-512-1	399	1	−8.6
hsa-miR-516b-2	130	1	−7.0
hsa-miR-548f-1	361	1	−8.5
hsa-miR-598	555	3	−7.5
hsa-miR-93	2340	377	−2.6
hsa-miR-96	174	2	−6.6

Numbers are normalized miRNA counts in each library.

**Table 2:** Upregulated miRNAs in RPE during ESC differentiation.

miRNA	H9 ESC	H9 RPE	log2(fold change)
hsa-let-7a-2	305	9107	4.9
hsa-let-7c	20	1288	6.0
hsa-let-7f-1	115	11339	6.6
hsa-let-7f-2	118	11685	6.6
hsa-let-7g	11	11615	10.0
hsa-let-7i	7	9875	10.5
hsa-miR-100	13	6309	8.9
hsa-miR-125a	37	19072	9.0
hsa-miR-125b-1	19	39452	11.0
hsa-miR-125b-2	18	38627	11.1
hsa-miR-139	5	91	4.2
hsa-miR-151b	10	57	2.5
hsa-miR-152	13	2087	7.3
hsa-miR-181d	1	122	6.9
hsa-miR-214	2	602	8.2
hsa-miR-22	25	2563	6.7
hsa-miR-222	167	1640	3.3
hsa-miR-23a	52	3188	5.9
hsa-miR-26a-1	636	6842	3.4
hsa-miR-26a-2	639	6848	3.4
hsa-miR-3120	2	602	8.2
hsa-miR-4521	16	156	3.3
hsa-miR-98	3	930	8.3

**Table 3:** RPE signature genes associated with downregulated miRNAs.

ADAM9	MYRIP
ANKRD12	NAV3
CDH1	NEDD4L
CDO1	PITPNA
CLCN4	PKNOX2
CRIM1	RAB38
DIXDC1	RHOBTB3
DMXL1	RPE65
ENPP2	RRAGD
FRZB	SDC2
GAS1	SGK3
ITGAV	SMAD6
ITM2B	SORBS2
MAB21L1	TIMP3
MBNL2	TRPM1
MET	TYRP1
MFAP3L	VEGFA

**Figure 3:**
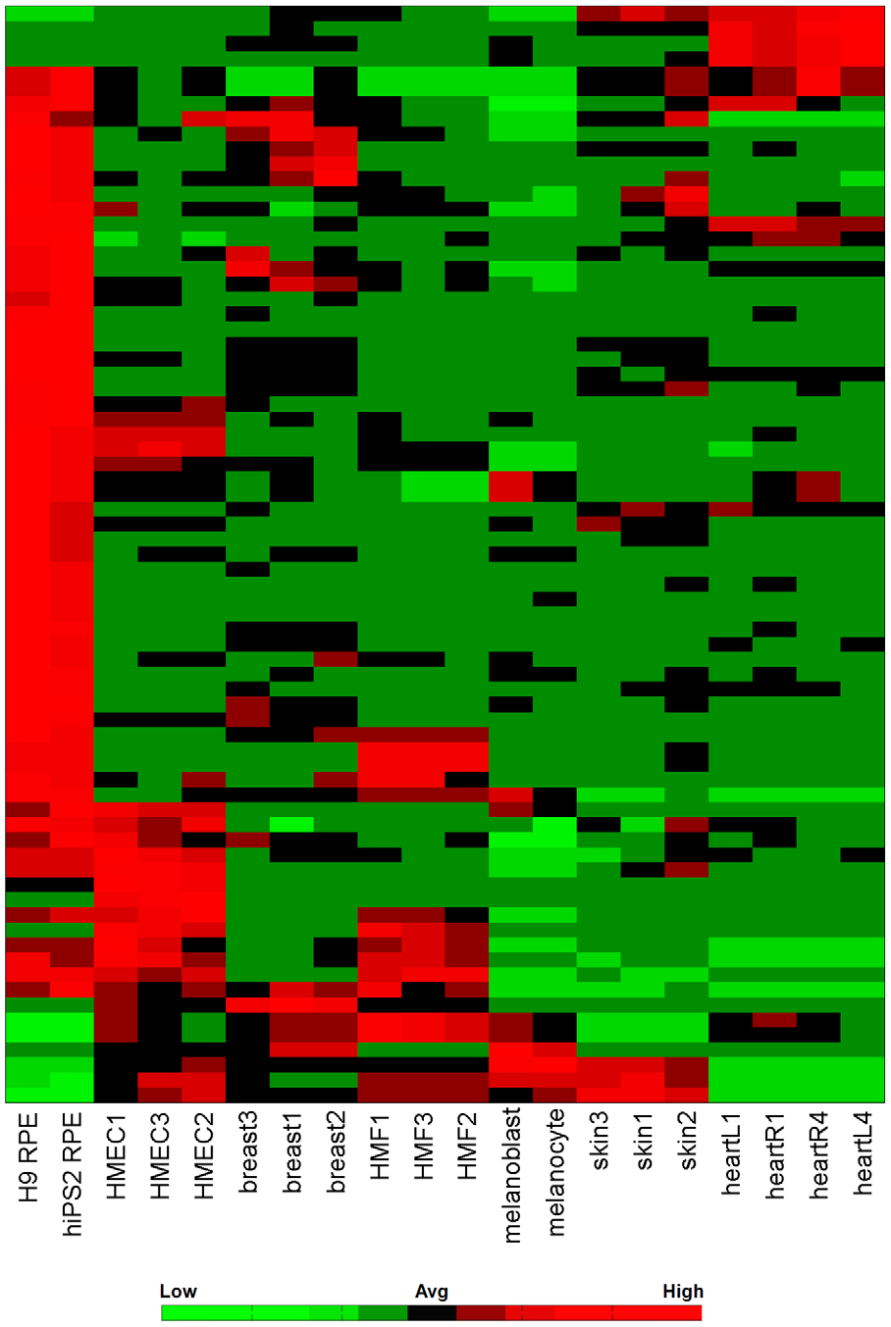
RPE-specific miRNA expression. Heatmap representing all miRNAs that are significantly up- or down-regulated compared to other somatic tissues.

**Figure 4:**
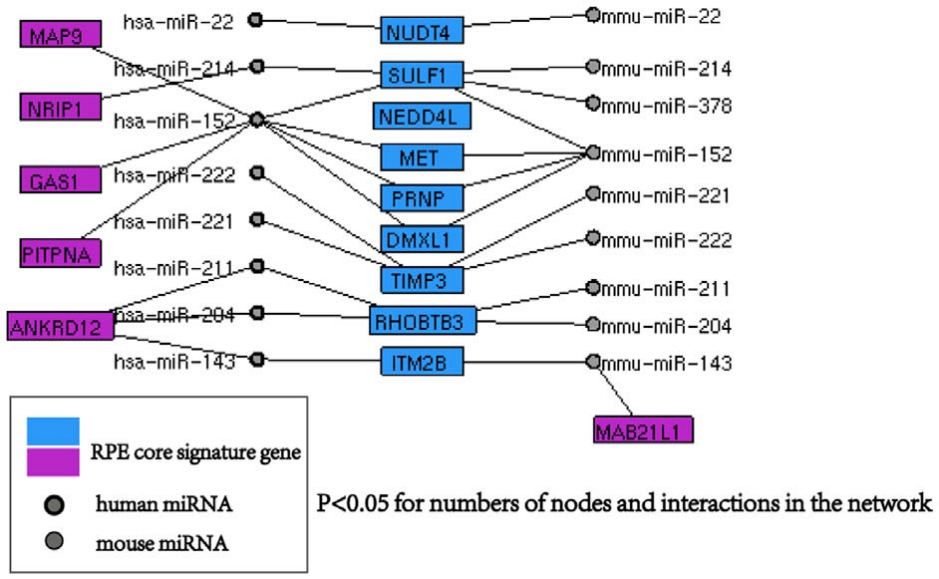
Human-mouse RPE miRNA interaction network for assembly of RPE signature genes. The network shows human-mouse RPE miRNA shared RPE signature genes, blue nodes are only targeted by one specie, purple nodes are shared by two species.

### MiRNA dynamics during RPE differentiation

To examine miRNA dynamics during RPE differentiation, we compared miRNA profiles during the course of RPE differentiation at four distinct stages: ES cells (stage 1), 15 days partially differentiated ES cells (stage 2), early pigmented clusters after 30-day differentiation (stage 3), and RPE (stage 4). We used one-way ANOVA test to determine whether miRNA expression within one group was significantly differentially expressed compared to the other three groups. In total, we found 21 and 49 miRNAs that were significantly up- and down-regulated in RPE compared to ES cells (*p*<0.01) (Figure 2, Table 1, 2). In particular, we found two groups of miRNAs that were dynamically regulated. One group of miRNAs was significantly and progressively up -regulated during differentiation from hESCs/iPSCs to 15 days differentiation, pigmented cluster, and all the way to fully differentiated RPE. Similarly, we found that another set of miRNAs was gradually down-regulated during the course of RPE differentiation (Figure 2A). As expected, the miRNAs we identified were mostly involved in differentiation. For example, miRNAs that promote differentiation such as let-7 family were up-regulated whereas miRNAs that promote pluripotency such as the miR-300 cluster are down-regulated. We were able to validate the expression of these miRNAs and others using Taqman qPCR, confirming our small RNA-Seq quantitation (Figure 2C). Thus, our data indicated that the majority of miRNA changes during RPE differentiation are involved in silencing of pluripotency-related miRNAs and activation of differentiation miRNAs common to most somatic cells. Interestingly, we also found a group of miRNAs that were specifically enriched at stage 2 and 3 of differentiation, but depleted in both stage 1and 4 (Figure 2B). KEGG pathway analysis of the predicted targets of these miRNA was mostly involved in endocytosis, active transport, lysosome and other RPE related function (Figure S1). These miRNA may be involved in early phase of hESC/RPE differentiation.

To examine the predicted targets of down-regulated miRNAs, we used two commonly used miRNA target prediction software TargetScan and miRanda. We compiled a list of non-redundant predicted targets, combined outputs from both softwares, and found several thousand putative targets. Gene Ontology (GO) analysis revealed predicted targets were enriched for biological processes such as gene expression, cell differentiation, and neuron differentiation (Figure S1). We next compared these targets with the list of 87 RPE signature genes [9] and found that 34 genes were predicted targets of the downregulated miRNA in RPE (Table 3), suggesting a reverse correlation between down-regulated miRNA and up-regulated RPE signature genes. Interestingly, RPE-specific gene RPE65 was a predicted target for the set of downregulated miRNAs in both softwares by different miRNAs, suggesting the involvement of miRNAs in regulating RPE65 expression during RPE differentiation. In short, our result predicts that some of the downregulated miRNA may be functionally relevant and contribute to the de-repression of key RPE signature genes.

### Tissue-specific MiRNA signature in RPE

Having identified the miRNA expression changes during RPE differentiation, we next focused on identifying the miRNA signature that distinguishes RPE from other somatic tissues. To identify the miRNAs that were exclusively expressed in human RPE, we compared mature RPEs (H9-RPE and hiPS2-RPE) with various somatic tissues. Using one-way ANOVA tests, we found 69 and 15 relatively enriched and depleted miRNAs in RPE (Figure 3). Notably, epithelial transition related miRNAs miR-204 and miR-211[19] were highly enriched in RPE compared to other tissues. These two miRNAs were specifically present in RPE but not in other epithelial cell types. Furthermore, miR-204/211 were also up-regulated during RPE development, underscoring the importance of miR-204/211 expression in RPE. Functional annotation of predicted targets for RPE enriched miRNAs revealed terms involved in a variety of biological processes. In contrast, putative targets of downregulated miRNAs was primarily associated with cell adhesion, notably in the focal-adhesion pathway. This result suggested that the most defining difference between RPE and other tissues was related to the epithelial characteristics of RPE. Finally, both upregulated and downregulated miRNAs have predicted targets significantly enriched in different components of the TGF-β pathway, implicating a role for the miRNA-mediated regulation of TGF-β pathway in RPE differentiation. Together, the tissue specific miRNAs we identified appear to be predominantly involved in maintaining the epithelium and TGF-β signaling.

### Conservation of regulatory network between human mouse RPE miRNA-signature genes

Because many core biological functions are conserved between human and mouse, we cross-referenced our data with a previously published mouse RPE miRNA profiling dataset [20] and used NAViGATOR [21] to analyze the complex relationships between miRNA and RPE core genes. We identified 9 conserved miRNAs that appeared in top 100 most abundant miRNAs in both the human and mouse RPE datasets. After comparing their predicted targets to 87 RPE signature genes, we obtained a network map between homologous miRNAs and human RPE signature genes (Figure 4). In this network, one miRNA can target several genes and one gene can be targeted by several miRNAs. We found 8 RPE signature genes were targeted by both human and mouse miRNAs, 5 genes were exclusively targeted by human miRNA and 1 gene is exclusively targeted by mouse miRNA. GO analysis of 8 shared genes has the same function both in human and mouse. Notably, 7 of these conserved miRNA are appear to be specifically expressed in RPE compared to other tissues, indicating expression of these key miRNAs is critical for defining RPE cells.

## Discussion

The RPE is a highly specialized epithelium that is crucial for maintaining photoreceptor function and visual cycle [1]. Previous studies have shown a critical role for miRNAs in eye development, function, and survival *in vivo* [16], [23]. Dicer-deficient mice show multiple defects, indicating an important role for miRNAs in many tissues including the eye [24], [25], [26]. Previous miRNA profiles in RPE have primarily focused on mouse RPE or the human cell line ARPE19 [20], [22], however, miRNA profiles in human RPE are still limited. MiRNAs are proposed to act as fine tuners of gene expression either through inhibiting translation in the cytoplasm, or promoting mRNA degradation in the nucleus [27], [28]. More recently, miRNAs have been shown to primarily repress genes through transcriptional control [29]. Therefore, we used different software to predict the mRNA target of differentially expressed miRNA. Many of the down-regulated miRNAs potentially target a generous portion of RPE signature genes. However, a recently published study comparing various miRNA prediction softwares show very low concordance between three major prediction algorithms (miRanda, TargetScan, PicTar), indicating the standard for predicting miRNA target genes are still not robust [30]. To the best of our abilities and available tools, we employed these prediction algorithms to provide putative targets of key miRNAs.

Through profiling miRNA expression at four defined time points during RPE differentiation, our study demonstrated that each stage of RPE differentiation has a unique subset of miRNAs that are significantly differentially expressed compared to all other stages. We identified miRNAs that were exclusively enriched in ESCs, intermediate stages of spontaneously differentiated ESCs, and fully derived RPE. In addition, we were able to show that a portion of the miRNA become gradually increased or decreased, or transiently increased during the differentiation process, suggesting the expression level of particular miRNAs may be used as an indicator for RPE maturation. Thus, our study indicated that the degree of RPE differentiation can be gauged by profiling the specific mRNA expression patterns during RPE differentiation as shown in Figure 2. Furthermore, by incorporating miRNA profiles from various somatic tissues, we were able to find a portion of miRNA that are specifically expressed in human RPE cells. Functional annotation of the predicted targets using DAVID revealed RPE-specific miRNAs are primarily associated in regulating the epithelial barrier and TGF-β pathway. TGF-β signaling pathway is essential for epithelium-mesenchymal transition in RPE cells, indicating suppression of the TGF-β pathway contributes to maintaining the epithelium in RPE [31], [32]. Future study shall validate the function of these miRNA in RPE differentiation through gain or loss of function experiments. We also find a portion of human RPE-specific miRNAs also share enrichment in mouse RPE, indicating a potentially conserved functional role for these miRNAs. We suggest that the human RPE-specific miRNA signature may serve as molecular markers for characterizing functional RPE.

Age-related macular degeneration (AMD) is characterized by malfunction and degeneration of RPE. Recently, Kaneko et al. [33] discovered a miRNA-independent cell survival function for DICER1 in the context of AMD pathology, demonstrating upregulation of Alu elements in the absence of DICER1 promoted RPE cell death. However, this does not exclude the possibility for miRNAs in maintaining other features of RPE. Indeed, other studies have shown that miRNA may play important roles in AMD pathogenesis. Lin et al. discovered that miR-23a was down regulated in AMD eyes while upregulation of miR-23a can protect RPE cell from oxidative damage in ARPE19 cells [34]. In our dataset, miR-23a was enriched in RPE cells, and its expression was significant increased during development, supporting the hypothesis that miR-23a expression is necessary for maintaining healthy RPE. Overall, our data uncovered a unique set of miRNAs that are expressed in RPE, suggesting a small number of key miRNAs may contribute to promoting RPE survival and function.

## Materials and Methods

### Differentiation of hESC/hiPSC-RPE

The human embryonic stem cell lines H9, HSF1 as well as induced pluripotent stem cell line hIPS2 and mouse embryonic feeder cells were obtained from the UCLA Stem Cell Core [9]. Human embryonic stem cell line BG01 was a generous gift from Dr. James Thomson [18]. Pluripotent stem cells (hESC and iPSCs) were plated onto gamma-rays irradiation mouse embryonic feeder cells with DMEM/F12 culture medium containing 20% Knock-Out Serum Replacement, 0.1 mM nonessential amino acids, 0.1 mM b-mercaptoethanol and 100 ng/ml zebrafish basic fibroblast growth factor (zfbFGF) on a 6-well plate. Briefly, cells were cultured at 37°C in 5% CO2 for 6–10 days after which zfbFGF was omitted to facilitate spontaneous cell differentiation.

Pigmented colonies were observed within 4 weeks and allowed to expand for a few weeks, with media changes every 2–3 days. Pigmented cells were enriched by manual dissection using insulin needle followed by seeded on growth factor reduced Matrigel(BD Biosciences, diluted 1∶30) coated plate and transwell membranes.

RPE medium were changed to support pigment cluster expansion [containing a-MEM, 1× N2 supplement(Gibco), 1× Non-essential amino acid solution, 250 mg/ml taurine, 13 ng/ml Triiodo thyronin (Sigma-Aldrich, Gillingham, UK), 20 ng/ml Hydrocortisone (Sigma), 2mM L-glutamine (Invitrogen, Paisley, UK), 1x Penicillin-streptomycin and 10% Hyclone heat-inactivated foetal bovine serum(Thermo Scientific, Northumberland, UK)], which was replaced daily.

After 2 to 3 months, under these conditions, hESC-RPE and hiPSC RPE would form monolayer sheets of pigmented cells that can be dissected for gene expression analysis.

### Small RNA isolation

The total RNA containing the miRNA species were extracted from pigment cluster and RPE cells using mirVana miRNA Isolation kit (Ambion). The RNA quality and yields were analyzed by gel electrophoresis and Nanodrop. RNA samples were then aliquoted and stored at −80°C for small RNA library construction.

### Construction of small RNA library and high-throughput sequencing

Total RNA (1∼10 μg) was ligated with a pair of Illumina adaptors to their 5′and 3′ends. After reverse transcription, the small RNA molecules were amplified using multiplex adaptor primers for 12 cycles and the fragments around 93∼100 bp (small RNA+adaptors) were then isolated from agarose gel. The purified DNA was used directly for cluster generation and sequencing analysis using the Illumina's HiSeq2000 sequencing according to the manufacturer's instructions. Then the image files generated by the sequencer were processed to produce digital-quality data. Reads were trimmed for the 3′ adapter, then mapped the hg19 genome using Bowtie [17]. We accepted only reads that had at most 1 mismatch to the hg19 genome and mapped to no more than 10 places in the genome; the exact parameters in Bowtie are –v1 –m10 – all – strata.

### Statistical analysis and bioinformatics

Data analysis was carried out using Matlab. Briefly, differential expression analysis between four differentiation stages and different somatic tissues was determined using a combination of one-way ANOVA, false discovery rate, and fold change. Scripts are available upon request. The gene targets of differentially expressed miRNAs were predicted by two miRNA target prediction algorithms miRanda (http://microorna.sanger.ac.uk/sequencs/), and Targetscan (http://www.targetscan.org/). We used a common cutoff of mirSVR score ≤−1.2 as a threshold to reduce the false-positive rate of predicted targets. Gene Ontology (GO) and KEGG (Kyoto Encyclopedia of Genes and Genomes) pathway analysis of predicted target genes of miRNAs was performed by using DAVID 6.7, a web-based application (http://david.abcc.nicfcrf.gov/home.jspand default parameters). The obtained BP and KEGG categories were filtered for FDR≤5 against the *Homo Sapiens* background. The relationship between candidate miRNA predicted to the affected mRNA and genes were constructed via NAViGATOR software.

### Quantification of mature miRNAs by real-time qRT-PCR

15μl reverse transcription reactions containing 1–10 ng total RNA, 0.5 mM each dNTP, 5U MultiScribe Reverse Transcriptase, 1xRT buffer, 2.5 mM dNTP, 4U RNase Inhibitor and nuclease free water. Reverse transcription reactions were incubated at 16°C for 30 min, 42°C for 30 min, 85°C for 5 min, then store at 4°C until use in TaqMan assays. 20 μl TaqMan real-time PCR reactions consist of 1× TaqMan Universal PCR Master Mix, 1× TaqMan miRNA assay, 1.33 μl of undiluted cDNA, and nuclease free water. Each TaqMan miRNA assay was done in duplicate for each sample tested. Relative quantities were calculated using the 2^−ΔΔct^ method with U6 snRNA as endogenous control. Reactions were run with the Standard 7300 default cycling protocol without the 50°C incubation stage, with reactions incubated at 95°C 10 min, followed 40 cycles of 95°C 15 sec, 60°C 1 min. Fluorescence readings were collected during the 60°C step.

### Accession number

The data generated for this work have been deposited in the NCBI Gene Expression Omnibus (GEO) (http://www.ncbi.nlm.nih.gov/geo/) and are accessible through GEO Series accession number GSE37686.

## Supporting Information

Figure S1 **Functional annotation from Gene Ontology analysis of miRNAs specifically expressed in human RPE cells.**
(DOC)

Table S1 **Gene expression data sets of various somatic tissues from GEO.**
(XLSX)
