# Supplementary material for: Identification of miRNA Signatures during the Differentiation of hESCs into Retinal Pigment Epithelial Cells
Source: PLoS One. 2012 Jul 27;7(7):e37224. doi: 10.1371/journal.pone.0037224 (PMC3407211; doi:10.1371/journal.pone.0037224)
Supplement: Figure S1 — Functional annotation from Gene Ontology analysis of miRNAs specifically expressed in human RPE cells. (DOC) [file pone.0037224.s001.doc]

Figure S1: Functional annotation from Gene Ontology analysis of miRNAs specifically expressed in human RPE cells. (A) Down-regulated miRNA in RPE during hESC differentiation. (B) Up-regulated miRNA in RPE during hESC differnetiaton.(C) Intermediate stage up-regulated miRNA in RPE during hESC differentiation. (D) Tissue-specific miRNA in RPE.


 
 
 
 
 
 
 
 
 
 
 
 
 
